# Alginate Core–Shell Capsules for 3D Cultivation of Adipose-Derived Mesenchymal Stem Cells

**DOI:** 10.3390/bioengineering9020066

**Published:** 2022-02-06

**Authors:** Sabrina Nebel, Manuel Lux, Sonja Kuth, Faina Bider, Wolf Dietrich, Dominik Egger, Aldo R. Boccaccini, Cornelia Kasper

**Affiliations:** 1 Institute of Cell and Tissue Culture Technologies, Department of Biotechnology, University of Natural Resources and Life Sciences BOKU Vienna, 1190 Vienna, Austria; sabrina.nebel@boku.ac.at (S.N.); manuel.lux@boku.ac.at (M.L.); dominik.egger@boku.ac.at (D.E.); 2 Institute of Biomaterials, Department of Materials Science and Engineering, University of Erlangen-Nuremberg, 91058 Erlangen, Germany; sonja.kuth@fau.de (S.K.); faina.bider@fau.de (F.B.); aldo.boccaccini@fau.de (A.R.B.); 3 Department of Gynecology and Obstetrics, Karl Landsteiner University of Health Sciences, Alter Ziegelweg 10, 3430 Tulln, Austria; wolf.dietrich@kl.ac.at

**Keywords:** mesenchymal stem cells, core–shell capsule, 3D cell cultivation technologies, cell expansion, alginate

## Abstract

Mesenchymal stem cells (MSCs) are primary candidates in tissue engineering and stem cell therapies due to their intriguing regenerative and immunomodulatory potential. Their ability to self-assemble into three-dimensional (3D) aggregates further improves some of their therapeutic properties, e.g., differentiation potential, secretion of cytokines, and homing capacity after administration. However, high hydrodynamic shear forces and the resulting mechanical stresses within commercially available dynamic cultivation systems can decrease their regenerative properties. Cells embedded within a polymer matrix, however, lack cell-to-cell interactions found in their physiological environment. Here, we present a “semi scaffold-free” approach to protect the cells from high shear forces by a physical barrier, but still allow formation of a 3D structure with in vivo-like cell-to-cell contacts. We highlight a relatively simple method to create core–shell capsules by inverse gelation. The capsules consist of an outer barrier made from sodium alginate, which allows for nutrient and waste diffusion and an inner compartment for direct cell-cell interactions. Next to capsule characterization, a harvesting procedure was established and viability and proliferation of human adipose-derived MSCs were investigated. In the future, this encapsulation and cultivation technique might be used for MSC-expansion in scalable dynamic bioreactor systems, facilitating downstream procedures, such as cell harvest and differentiation into mature tissue grafts.

## 1. Introduction

Adipose-derived mesenchymal stem cells (adMSCs) are primary candidates in tissue engineering and stem cell therapies due to their intriguing regenerative potential, immunomodulatory effects [1,2,3], and availability from different sources [4]. The majority of cell-based therapies however require large numbers of cells to reach clinical relevance (approximately 1–2 × 10^6^ cells per kilogram of body weight), making large-scale ex-vivo expansion inevitable. In recent years, research has transitioning from 2D monolayer cultivation of MSCs on plasticware towards three-dimensional (3D) cultivation, as it provides the cells with a more relevant physiological environment. For example, their ability to self-assemble into 3D aggregates under dynamic cultures was shown to improve their therapeutic properties, including maintenance of stemness, differentiation potential, secretion of cytokines, and homing capacity after administration [5,6]. 

However, large-scale expansion systems, such as commonly available stirred tank reactors, inflict high shear forces on the cell constructs and the resulting mechanical stress can decrease their regenerative properties [7,8]. In particular, upscaling to larger cultivation volumes requires increased mixing speeds to eliminate nutrient gradients and reach sufficient gas exchange [7,9]. However, cells that are completely embedded in a polymer matrix lack cell-to-cell interactions found in their physiological environment [10]. Moreover, monitoring and recovery are considerably more difficult than in their 2D counterparts [11]. 

One approach to create a protected microenvironment for cells without restricting their interaction, is core–shell encapsulation. Such capsules consist of an outer barrier made from e.g., sodium alginate [12,13,14,15], poly-L-lysine (PLL) [15,16] or cellulose [15,17] which allows for nutrient and waste diffusion and an inner compartment for direct cell-cell interactions. This method has been used for cultivation of a multitude of different cells by now. For example, Siltanen et al. dynamically expanded HepG cells in PEG core–shell capsules as liver analogues for cell-based therapies or in-vitro models for drug development [18], Alessandri et al. and Yu et al. have used core shell capsules to investigate tumor growth [19,20]. In particular, alginate-based capsules are of interest as delivery systems for cell-based therapies [16,21,22], as a protective layer to prevent transplant rejection. Core–shell encapsulation can also serve as a high-throughput method for controlled generation of spheroids [15]. Interestingly, in the context of cell expansion and large(r)-scale production processes, this method was recently used by Cohen et al. and Fattahi et al. to cultivate human pluripotent stem cells, with striking results of up to 282-fold expansion [12,23].

However, to our knowledge so far, no efforts have been made to establish a core–shell encapsulation procedure for large-scale expansion of MSCs ex-vivo.

Here, we present a “semi scaffold-free” approach to protect the cells from high shear forces by a physical barrier while allowing them to form a 3D structure with in vivo-like cell-to-cell contacts. We highlight a relatively simple method to create core–shell capsules by inverse gelation. Contrary to other encapsulation approaches, where multiple crosslinking, coating and core liquification steps are often necessary, as well as expensive equipment [24], we established a robust workflow using inverse gelation of sodium alginate to create core–shell capsules. In applications where alginate beads are formed, a cell-containing alginate solution is extruded into a crosslinking bath containing divalent cations, most often in the form of CaCl_2_. For inverse gelation, the cell suspension contains the cations instead and is extruded into a stirred alginate bath. Upon submersion of the liquid droplet in the alginate bath, ionotropic gelation from the droplet surface outward takes place, leaving a liquid core.

Our aim was to establish an optimal protocol for encapsulation of human adipose derived MSCs as well as to evaluate their in-vitro viability and proliferation capacity within the core–shell capsules. In the future, this liquid-core encapsulation technique might be used for expansion of MSCs in scalable and dynamic bioreactor systems. Overall, this cultivation technique could help to improve mesenchymal stem cell expansion, production of cell-based therapeutics, such as extracellular vesicles and growth factors, and facilitate downstream procedures, such as cell harvest and differentiation into mature tissue grafts.

## 2. Materials and Methods

If not otherwise stated, reagents were purchased from Sigma Aldrich, St. Luis, MO, USA.

### 2.1. Cell Culture

Human adipose derived MSCs (adMSCs) were isolated from female donors, from skin flaps removed during routine re-laparotomies, e.g., caesarian sections. Isolation from human tissue was approved by the ethics committee of Scientific Integrity und Ethics of the Karl Landsteiner University of Health Sciences). All donors gave written consent. The donor tissue was stored at 4 °C and processed within 24 h after surgery. Briefly, fat tissue was separated from the skin flap, minced with scissors, and digested with collagenase type IA for 1 h. After several centrifugation and washing steps, the stromal vascular fraction was released in a cell culture flask and adMSCs were selected by plastic adherence. After isolation, adMSCs were cultivated in standard medium composed of MEM alpha (Thermo Fisher Scientific, Waltham, MA, USA), 0.5% gentamycin (Lonza, Basel, Switzerland), 2.5% human platelet lysate (hPL, PL BioScience, Aachen, Germany), and 1 U/mL heparin (PL BioScience, Aachen, Germany) in a humidified incubator at 37 °C and 5% CO_2_. Cells were cryo-preserved in αMEM, 2.5% hPL, 10% DMSO (Sigma Aldrich, St. Louis, MO, USA), and 1 U/mL heparin in a liquid nitrogen tank. After thawing, the cells were expanded for two passages in cell culture-flasks (Sarstedt, Nümbrecht, Germany) and harvested using Accutase (GE healthcare, Little Chalfont, UK).

### 2.2. Core–Shell Capsule Production

Four thickening agents, polyethylene glycol 6000 PEG6000 (MERCK, Hohenbrunn, Germany), maltodextrin, carboxymethylcellulose (Akzo Nobel Chemical, Amersfoort, Netherlands), and xanthan gum were UV-sterilized and resuspended in αMEM at 20%, 66.6%, 1.5%, and 0.3% *w/v*, respectively. PEG6000, maltodextrin, CMC, and xanthan gum were used to increase the viscosity of the suspension to ensure spherical shape of the capsules, the concentrations were taken from literature [25,26,27].

The different viscous media solutions were mixed with a 13% CaCl_2_ stock solution (pH 7.4) to a final concentration of 1.3% CaCl_2_, drawn up into a 1 mL syringe (Fisher Scientific GmbH, Schwerte, Germany) and extruded into a rapidly stirred sodium alginate (Sigma Aldrich, St. Louis, MO, USA) bath. After shell-crosslinking, the alginate solution was diluted with PBS (Gibco, Thermo Fisher Scientific, Waltham, MA, USA), before capsules were collected and transferred into PBS. After this washing step, the capsules were incubated in a 1.3% CaCl_2_ bath for 2 min. After 2 subsequent PBS washing steps, capsules were collected in 6-well plates (Sarstedt, Nümbrecht, Germany) for further analysis.

### 2.3. Cell Encapsulation in Core–Shell Capsules

Cells were cultured as described in Section 2.1 before they were encapsulated into alginate capsules. The process of capsule production remained the same as described in Section 2.2, with the difference that cells were resuspended in complete cell cultivation media (αMEM, 0.5% gentamycin, 1 U/mL Heparin and 5% hPL) supplemented with one of the 4 thickening agents, respectively, before mixing with CaCl_2_ and extrusion into the alginate solution. As controls, capsules without cells (blanks) were prepared as well and treated the same as those containing cells.

### 2.4. Determination of Capsule Dimensions

Light microscopic images were taken using a Leica DMi1. At least 10 images per sample type were processed manually in FIJI (ImageJ) [28]. Measurements taken included the inner and outer diameter of capsules and wall thickness.

### 2.5. Mechanical Testing

Mechanical properties were measured by parallel plate compression (MicroTester LT, CellScale, Canada). The samples were measured in a bath of α-MEM, supplemented with 1% gentamycin, at 37 °C to simulate culture conditions. Cyclic measurements consisted of 6 cycles with a maximum compressive force of 2.5 mN. Stress-strain curves were obtained by converting the measured force-displacement data. Young’s moduli were calculated using a modified version of the Reissner model for capsules with shell thicknesses larger than 1/20 of the capsule radius (see Equation (3) in Section 3.4) [29].

### 2.6. Diffusion Characteristics

Capsules were incubated in 18-well µ-Slides (ibidi, Gräfelfing, Germany) with αMEM containing 0.25 mg/mL fluorescein isothiocyanate (FITC)-labelled dextran, Mw ~4 kDa and assessed at different time points by confocal microscopy (Leica TCS SP8-STED). Images were captured at 488/520 nm (Ex/Em) to monitor dextran diffusion into the capsule interior. Mean intensity of bulk volume, shell and inner capsule core was determined using Las X (Leica) and FIJI software and a ratio of internal to external fluorescence was calculated. Results are presented as ratios of the internal to external fluorescence over time.

### 2.7. Metabolic Activity

Metabolic activity was measured using a resazurin-based assay “TOX8” purchased from Sigma-Aldrich. The 2D experiments were performed according to the manufacturer’s instructions. For measurements in 3D capsule cultivation, capsules were transferred to 2 mL reaction tubes (Brand Scientific GMBH, Wertheim, Germany), weighed, and 300 µL of TOX8 working solution per 100 mg capsules were added. Capsules without cells were used as the blank control. The tubes were placed on a shaker at 100 RPM for 3 h before measurement of the supernatant. Fluorescence intensity was measured at 560/590 nm Ex/Em using a Tecan infinite M1000. Values presented are blanked and normalized to d0 samples.

### 2.8. Cell Harvest

Cells were retrieved from the capsules for determination of cell numbers. For this, capsules were collected in 15 mL reaction tubes (Greiner Bio-One, Kremsmünster, Austria) and weighed. About 300 mg per sample were collected, the alginate shell was dissolved with 100 mM sodium citrate pH 7.4 for 2 min at room temperature. Before centrifugation at 500× *g* for 5 min sodium citrate was diluted with 5-fold volume culture medium. Supernatant was removed and the cell pellet was resuspended in 200 µL Accumax™ and incubated for 30 min at 37 °C and 100 RPM. Afterwards, Accumax™ was inactivated by 3-fold the volume of cell culture medium. After centrifugation at 500× *g* for 7 min cell pellet was resuspended in fresh medium and counted manually using Neubauer counting chambers and Trypan Blue exclusion assay. Results are presented as both the number of cells/capsule and number of cells/g capsules.

### 2.9. EdU Assay

To visualize cell proliferation in the capsules, a commercially available kit, EdU Click 488, was used (baseclick GmbH, Munich, Germany). Capsules were incubated with EdU for 48 h (d2–4) at a concentration of 10 µM. Afterwards they were fixed in 4% paraformaldehyde for 24, washed with PBS and stored in αMEM at 4 °C. The residual staining procedure was performed according to manufacturer’s instructions.

### 2.10. Live/Dead Staining

Capsules were stained with Calcein AM (Invitrogen, Thermo Fisher Scientific, Waltham, MA, USA) and Propidium Iodide (Invitrogen, Thermo Fisher Scientific, Waltham, MA, USA) at a final concentration of 1 µg/mL and 3.3 µg/mL, respectively, to visualize live and dead cells. The capsules were incubated for 30 min at 37 °C in the dark and transferred to fresh αMEM for fluorescence microscopic analysis.

### 2.11. Statistical Analysis

All quantification data are presented as mean ± the standard deviation (SD) with at least three independent replicates, the sample size “*n*” of the experiment is given in the legend of each corresponding figure. Data were plotted and analyzed using GraphPad Prism 6, significance is indicated as follows: * *p* < 0.05, ** *p* < 0.01, *** *p* < 0.001, **** *p* < 0.0001.

## 3. Results

### 3.1. Tuning Viscosity of Capsule Interior

**Figure 1:**
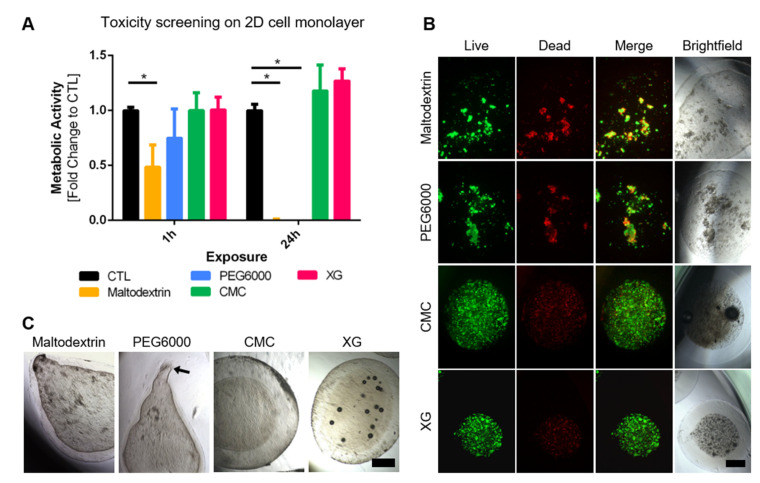
(**A**) Toxicity screening of thickening agents; adherent MSCs in 2D monolayer culture were exposed for 1 and 24 h to culture medium containing thickening agents at above stated concentrations; measurement of metabolic activity revealed significant decrease in maltodextrin and PEG6000 groups in comparison to controls treated with standard cell culture medium (*n* = 4, multiple *t*-test analysis, significance determined using Holm–Šídák method, α = 0.05); (**B**) live/dead staining of encapsulated MSCs 24 h after encapsulation; irregular capsule shells and cell clumps with high number of dead cells in PEG6000 and maltodextrin samples, good distribution, and high number of viable cells in CMC and XG samples (scalebar = 500 µm); (**C**) representative brightfield images of blank capsules produced with 4 different thickening agents (maltodextrin—66.6%, polyethylene glycol PEG6000—20%, carboxymethylcellulose CMC—1.5%, xanthan gum XG—0.3%), first two resulted in “tadpole”-shaped capsules, often not completely closed (black arrow), whereas last two showed good results (scalebar = 500 µm).

In order to increase the core viscosity, four different thickening agents were screened. Maltodextrin, polyethylene glycol (PEG6000), carboxymethylcellulose (CMC), and xanthan gum (XG) are widely used in food biotechnology but have previously been used in capsule production [25,26,27]. The concentrations initially tested were derived from the literature [25,26,27] and were 66.6%, 20%, 0.3%, and 1.5%, respectively. The initial screening consisted of producing capsules without cells to solely look at the geometry of the capsules, and a cytotoxicity test of the compounds in parallel. Additionally, cells were then encapsulated using the four thickening agents. The blank capsules produced can be seen in Figure 1, with maltodextrin and PEG6000 despite high *w*/*v* % being non-spherical, but rather “tadpole”-shaped and a majority not even completely closed (black arrow). CMC and XG however produced closed, spherical to slightly elliptical capsules. For the toxicity screening, adherent MSCs were exposed to thickening agent-supplemented medium for 1 or 24 h followed by measuring their metabolic activity. Cells exposed to standard cultivation medium served as control. After 1 h just slight differences can be seen between the control to the exposed cells, however results are significant for the longer exposure, with a significant decrease in signal of PEG6000 and maltodextrin containing samples.

Both results could also be seen whilst encapsulating MSCs, resulting in irregular capsules containing cell clumps with high number of dead cells (see Figure 1B). CMC and XG capsules however presented regular shapes and a high number of viable, well distributed cells inside. Therefore, these two thickening agents were deemed as suitable and used for further optimization and establishment of the cultivation protocol.

### 3.2. Encapsulation Procedure

**Figure 2:**
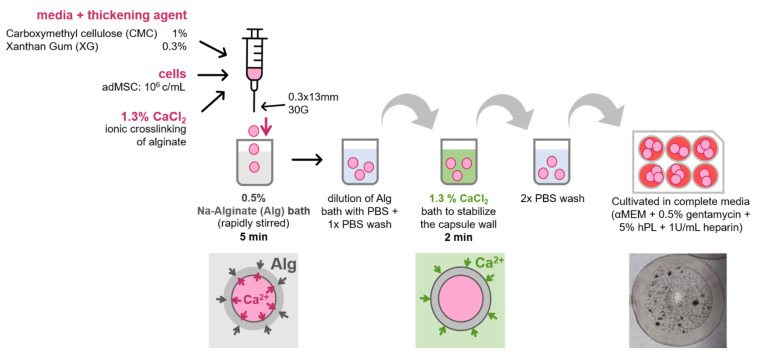
Schematic overview of optimal capsule production workflow.

Using the two thickening agents, the cell encapsulation protocol was optimized as follows:

Core–shell capsules were prepared by extrusion of a cationic solution into a sodium-alginate bath, a so-called inverse gelation. Carboxymethylcellulose and xanthan gum were UV-sterilized and resuspended in complete cell cultivation media (αMEM, 0.5% gentamycin, 1 U/mL Heparin and 5% hPL) at 1.11% and 0.333% *w*/*v*, respectively. AdMSCs were detached as described in Section 2.1. One million cells were resuspended in 900 µL of either one of the two viscous media solutions. The cell solution was then mixed with a 13% CaCl_2_ stock solution (pH 7.4) to a final concentration of 1.3% CaCl_2_, 10^6^ cells/mL and 1% *w*/*v* CMC or 0.3% XG, respectively. The solution was shortly spun down to remove air bubbles, drawn up into a 1 mL syringe and carefully extruded through a 30 G needle into a 0.5% *w*/*v* sodium alginate bath stirred at 400 RPM at 37 °C. A dropping height of 3 cm was used to prevent deformation of the droplets. Instantly after submersion of the CaCl_2_ suspension droplets in the alginate solution, a shell is formed by ionic crosslinking from the droplet surface outwards, leaving a liquid core. After 5 min under stirring, the alginate solution was diluted with the same amount of PBS to prevent individual capsules from sticking to each other, before the stirrer was stopped and capsules were collected using a fine sieve and transferred into PBS. After this washing step, the capsules were incubated in a 1.3% CaCl_2_ bath for 2 min to stabilize the outer shell. After two subsequent PBS washing steps, capsules were collected and transferred to 6-well plates containing 2 mL/well complete cell cultivation medium. The plates were placed at 37 °C, humidified atmosphere and 5% CO_2_ on a shaker (100 RPM) for the rest of the cultivation. Great care was taken to minimize the time from cell detachment until finished capsule production below 40 min, detaching cells in batches if necessary. A schematic of the procedure is depicted in Figure 2.

### 3.3. Assessment of Capsule Size and Shape

Thirty capsules each were imaged and analyzed to determine the mean size of their outer diameter, inner diameter, and shell thickness. For non-spherical capsules, the following equation was used to determine the average diameter *d_A_*:

(1) $$d_{A}=\sqrt[{3}]{lb^{2}},$$

where *l* denotes the length and *b* the width of the capsule [30].

**Figure 3:**
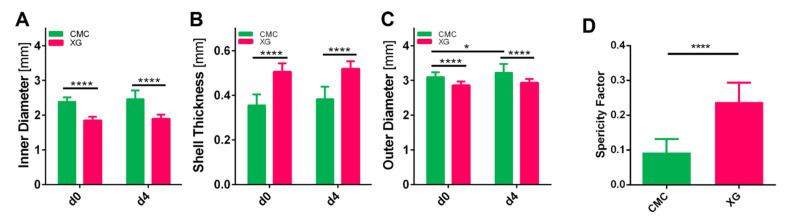
(**A**–**C**) Measurements of alginate capsule dimensions produced with CMC or XG: inner diameter (**A**), shell thickness (**B**), and outer diameter (**C**), measured on days 0 and 4 (*n* = 30, two-way ANOVA, significance determined using Tukey’s multiple comparisons test, α = 0.05); (**D**) sphericity factor (see Equation (2)) calculated for capsules (*n* = 30, students *t*-test, α = 0.05).

Capsules produced with a CMC core had an outer diameter of 3.10 ± 0.13 mm and an inner diameter of 2.39 ± 0.12 mm directly after casting, which were both significantly larger than those of the produced XG capsules, with an outer diameter of 2.86 ± 0.11 mm and inner diameter of 1.85 ± 0.11 mm (see Figure 3). Although the same Ca^2+^ and alginate concentrations were used, which have been shown to determine shell thickness [31,32], XG samples presented a significantly thicker shell of 0.51 ± 0.04 mm compared to 0.36 ± 0.05 mm for CMC. Alginate has previously been reported to swell during cultivation; therefore, we compared the sizes of the capsules directly after production and 4 days at culture conditions (100 RPM, 37 °C, 5% CO_2_). No significant change could be found in the shell thickness, however the total size of the CMC capsules increased slightly (from 3.10 ± 0.13 to 3.23 ± 0.25 mm). This could stem from a combination of swelling from both the core and shell combined.

Next to the size, shape was also analyzed, by calculating a sphericity factor (SF). The following formula was used to determine *SF*:

(2) $$SF=\frac{\left(l-b\right)}{\left(l+b\right)},$$

where *l* denotes the length and *b* the width of the capsule; therefore, the factor equals 0 for a perfect sphere and approaches 1 the more elongated [31]. Both cell suspension formulations resulted in ellipsoid capsules, CMC however showed a lot less difference in the longest to the smallest diameter with a sphericity factor of 0.096 ± 0.038, whereas XG capsules were perceivable elongated (SF = 0.24 ± 0.05).

### 3.4. Capsule Characterization

For mechanical characterization, individual capsules were compressed between two parallel plates to a maximum force of 2.5 mN and released back. Six loading and unloading cycles were performed and can be seen in a dimensionless approach, plotted as F/r_0_ and relative deformation δ to account for size differences of capsules (where: F = compression force, r_0_ = calculated radius of capsule according to Equation (1), δ = current distance/initial distance between compression plates).

**Figure 4:**
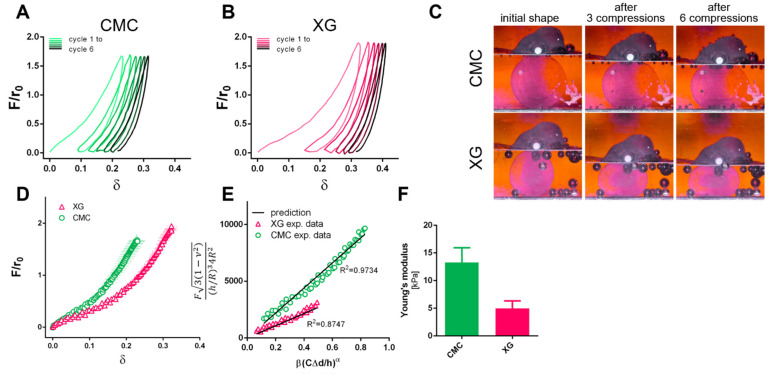
Dimensionless force-displacement curves for 6 loading and unloading cycles of CMC (**A**) and XG alginate capsules (**B**); (moving average, *n* = 3), (**C**) representative images of capsules during cyclic compression; the initial shape as well as the permanent deformation after multiple compressions can be seen; (**D**) dimensionless force-displacement curves of CMC and XG capsules (*n* = 3); (**E**) comparison of experimental data to theoretical prediction according to Equation (3) from which (**F**) Young’s modulus of the capsules was determined.

A viscoelastic behavior was recorded in both CMC and XG containing samples, as well as a permanent deformation after unloading (see Figure 4A–C) In Figure 4D, loading curves of both capsule types are shown. Here, it is evident that to achieve the same deformation, less force is necessary in the XG capsules. The Young’s moduli of the capsules were determined using a modification of Hertz and Reissner theory for capsules by Berry et al. [29] (see Equation (3)) in the range of 5–20% deformation (Hertz theory valid up to 30% deformation in alginate [33]):

(3) $$\frac{F\sqrt{3\left(1-\nu^{2}\right)}}{{\left(h/R\right)}^{3}4R^{2}}=E\beta {\left(\frac{C\delta_{plate}}{h}\right)}^{\alpha }\;$$

where *F* is the compressive force, *ν* is the Poisson’s ratio, *h* the shell thickness, *R* the capsule radius, *E* the Young’s modulus, *δ_plate_* is the displacement of the compression plate, *C*, *α*, *β* are fitting parameters to correct for shell thickness ratio, indenter shape and *ν*.

Values below 5% deformation were excluded due to measurement uncertainty at these small forces. Young’s moduli were determined for each individual capsule to determine the correct fitting parameters and were compared to the theoretical prediction. For CMC and XG capsules, R^2^ of 0.98 ± 0.02 and 0.80 ± 0.08 were determined, representative plots can be seen in Figure 4E. The difference in the curves is also reflected in the determined Young’s moduli of 13 ± 3 kPa and 5 ± 1 kPa for CMC and XG samples, respectively (Figure 4F).

**Figure 5:**
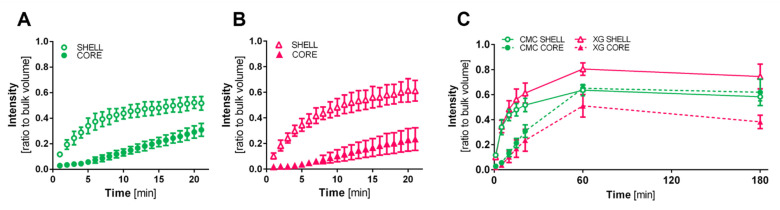
Diffusion of 4 kDa FITC-labelled dextran into shell and core of alginate capsules at 20 °C; (**A**) CMC containing capsules and (**B**) XG containing capsules in the first 20 min, presented as ratio of average fluorescence intensity of shell and core region to bulk volume intensity; for both conditions an initial increase in the shell material that starts to flatten after 5 min; diffusion into the core is delayed for both conditions, for the time it takes for the dextran to pass through the shell material, (*n* = 3); (**C**) diffusion properties over 3 h; core and shell of CMC capsules reach a common plateau of ~60% compared to bulk intensity already after 60 min; although the alginate shell of XG containing capsules shows intensity values between 70 and 80% for their plateau, the core region did not reach the same intensity after 3 h.

Diffusion properties of the capsules were investigated by incubation in 0.5 mg/mL FITC-labelled dextran (MW4000Da) for up to 3 h at 20 °C. Confocal microscopic images were taken, and the fluorescence intensity of the shell and capsule core was compared to the bulk fluorescence intensity. Initially, one can see a strong increase of fluorescence that flattens within the first 20 min in the shell, accompanied by a delay of the signal in the core (Figure 5). This marks the time needed for the dextran to pass through the shell. For the CMC capsule conditions, the saturation in the shell and core reach a common plateau of fluorescence of approximately 60% after 1 h and stay at similar intensity values until 3 h. For the XG capsules, although the shell reaches a plateau of above 70%, the diffusion to the core flattens at 60 min around 50% and does not further increase but sinks below 40% until hour 3. Generally, diffusion below 40–70 kDa has been shown previously for alginate-based solid beads [34,35,36], but a slightly better diffusion into the core is seen in capsules with CMC used as a thickening agent in this approach.

### 3.5. Recovery of adMSCs from Core–Shell Alginate Capsules

**Figure 6:**
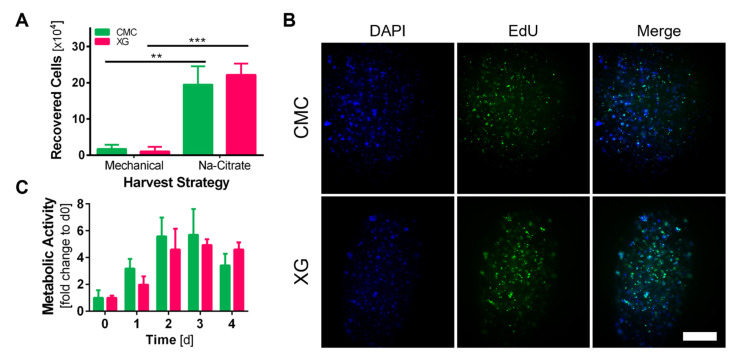
(**A**) Number of cells recovered from 1 g of capsules using different harvesting approaches; capsules were either mechanically disrupted, cell aggregates enzymatically disassociated and passed through cell strainer to remove alginate shell fragments or dissolved by addition of 0.1 M Na-citrate followed by enzymatical cell aggregates disassociation (*n* = 3, students *t*-test, α = 0.05); (**B**) representative images of EdU proliferation staining between d2 and d4 of cultivation, blue—DAPI: stains all nuclei, green—EdU-488: stains nuclei of proliferating cells, in both groups proliferating cells after initial compaction phase were detected (scale bar = 500 µm); (**C**) metabolic activity of encapsulated adMSCs measured daily, an initial increase can be seen until day 2 where values stayed constant (XG) or decreased (CMC) towards day 4 (*n* = 6, normalized to d0).

Two different approaches were compared to recover cells from capsules after 3 days of cultivation, to allow for cell aggregation. The shell was either mechanically disrupted by pipetting up and down, followed by enzymatic digestion of the multicellular aggregates. After 30 min of incubation, the suspension was passed through a cell strainer to remove alginate shell fragments. The other approach used 0.1 M Na-citrate to dissolve the alginate shell. After centrifugation and removal of the supernatant, here, cell aggregates were enzymatically dissociated before counting cells (Figure 6A). The number of cells recovered from capsules was significantly higher (CMC: 19.48 ± 5.05 XG: 22.19 ± 3.09 × 10^4^ cells) when the shell was dissolved with Na-citrate than when just mechanical processes were used (CMC: 1.73 ± 1.17 XG: 1.05 ± 0.23 × 10^4^ cells). Alginate shell fragments clogged the cell strainer in the mechanical approach, so only a small amount of the initial cell suspension could pass through, thus explaining the low recovery number. Therefore, the approach using sodium citrate was chosen for all further experiments.

### 3.6. Evaluation of Encapsulated adMSC Proliferation and Viability

Metabolic activity levels of encapsulated adMSCs were monitored daily for 4 days of cultivation. For this, an adapted resazurin assay was used. Capsules were transferred to 2 mL reaction tubes and weighed before adding 300 µL of resazurin working solution (prepared according the to manufacturer’s instructions) per 100 mg capsules. Afterwards, samples were incubated at 37 °C 100 RPM for 3 h before fluorescence signal of supernatant was measured at Ex/Em 560/590 nm. The adjustment to capsule weight was necessary to account for resazurin reagent dilution by the liquid contained in the capsules. Interestingly, in both groups, the signal shows a four–five-fold increase until days 2–3 but stays at that level or even decreases until day 4 (Figure 6C). There have been reports of MSCs cultured in spheroids attenuate proliferating after compacting [37,38]. Therefore, to investigate the proliferative behavior of the aggregated cells after this initial compaction phase, we performed EdU proliferation staining on days 2 and 4. It can be seen in Figure 6B that cells kept on dividing, thus incorporating EdU into their nuclei. Proliferating cells could be detected, distributed throughout the whole capsule interior and not confined to the outer core region. Further, not only did single scattered cells proliferate, but also if they were part of larger cell aggregates, as seen by overlay with nuclear counterstain (DAPI).

**Figure 7:**
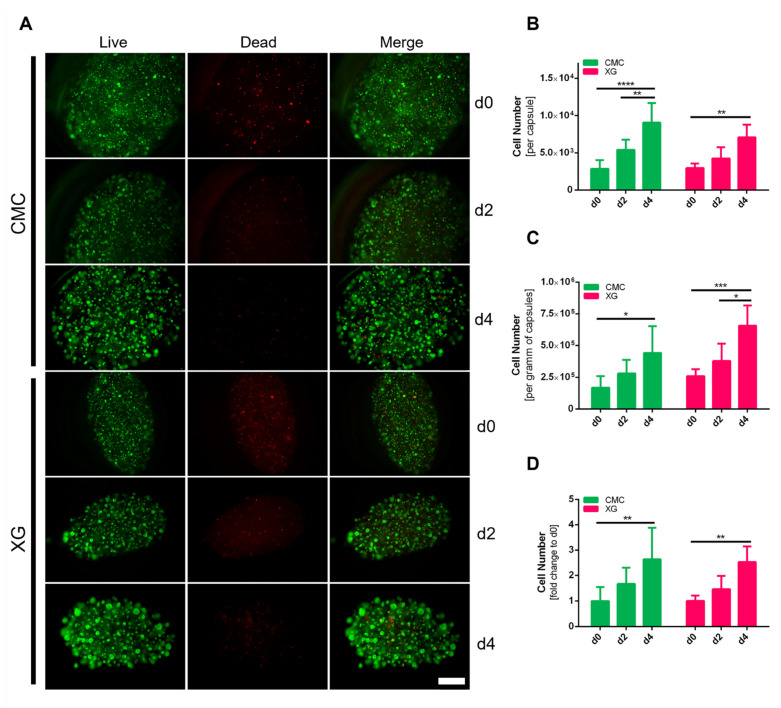
(**A**) Live/dead staining of encapsulated adMSCs during cultivation, green—Calcein AM: viable cells, red—propidium iodide: dead cells, high viability can be seen in all groups over the course of the cultivation, no necrotic core towards the center of the capsule cores are visible, initially mostly single cells distributed within capsule interior, formation of a multitude of multicellular aggregates visible, more pronounced in XG capsules (scale bar = 500 µm); (**B**–**D**) numbers of harvested adMSCs (**B**) per capsule, (**C**) per g of capsules or presented as fold change to day 0 of total cell number (*n* = 6, two-way ANOVA, significance determined using Tukey’s multiple comparisons test, α = 0.05).

Live/dead staining (Figure 7A) was performed directly on the day of cell encapsulation (d0), days 2 and 4. Whilst on day zero mostly single cells evenly distributed in the capsule core are visible, towards the end of cultivation a multitude of multicellular aggregates can be seen, slightly more pronounced in the XG-containing core. Only a small number of dead cells, stained by propidium iodide, were visible throughout the cultivation period. Slightly more on day 0, which might be caused by the stress on the cells during the encapsulation procedure. Overall cells are evenly distributed throughout the whole capsule no increase of dead cells toward the capsule core was detected, which is a problem often reported in 3D cell cultivation set ups [39].

To determine the actual cell numbers within the different capsules, we also recovered them using the previously described method using Na-citrate to dissolve the shell material. The cell number within the capsules increased significantly for both types. Although no significant difference could be determined between CMC and XG as internal matrix on day 4, with 9.05 ± 2.65 × 10^3^ cells/capsule slightly more could be recovered from CMC capsules compared to 7.07 ± 1.69 × 10^3^ cells/capsule from XG ones (Figure 7A). This is not surprising, as previously determined, the capsules differ significantly in size. Therefore, as is common with microcarriers, the number of cells per gram of capsules can be seen in Figure 7B. Moreover, here only a trend and not statistically significant differences between CMC (4.43 ± 2.10 × 10^5^ cells/g capsule) and XG capsules (6.56 ± 1.60 × 10^5^ cells/g capsule) can be seen, however now in favor of XG as thickening agent. Looking at the overall fold change of cell numbers throughout the four days of cultivation, also considering the production yield of capsules per mL of cell suspension (CMC: 3.83 ± 0.79 XG: 3.12 ± 0.30 g capsules/mL cell suspension), the cells within the two capsule types presented a 2.6-fold (2.64 ± 1.25) and 2.5-fold (2.53 ± 0.62) change for CMC and XG, respectively. No clear advantage could be quantified for one or the other core materials, reinforcing that both thickening agents are suitable for encapsulation of adMSCs in this approach.

## 4. Discussion

The aim of this study was to find a method to encapsulate MSCs into alginate core–shell capsules that can be used for scalable and automatable expansion. The main goal was for the technique to allow for high cell viability and proliferation of encapsulated cells as well as a high cell recovery and capsule characteristics suitable for dynamic cultivation.

During our initial screening for a suitable thickening agent to create the desired capsule shapes, we saw spontaneous formation of multiple cellular aggregates within the capsules rather than formation of just one aggregate. This behavior had been reported before by Mineda et al. [40] and Park et al. [41] within a non-crosslinked hyaluronic acid (HA) hydrogel. HA is a carbohydrate polymer formed of disaccharide subunits, which can be found especially in connective tissues, such as in skin and joints [42]. Overall, it compromises a major part of the extracellular matrix and can be found in almost all human tissues. Because of its role in vivo, it has been used for various medical applications [43,44,45]. CMC and XG are also polysaccharides, and all three of them are able to form hydrocolloids [46,47], but different from a lot of commonly used hydrogels, they are not crosslinked in our application presented in this paper. Interestingly, although CMC, a cellulose derivative produced from wood, and xanthan gum, a polysaccharide produced by bacteria, do not have a physiological role in mammalians, they still promoted spheroid formation of MSCs. In the scope of our work the use of these two compounds brings multiple advantages: firstly, they are both widely available and less expensive than HA. Secondly, as they promote in situ formation of multiple smaller spheroids in the capsule interior, the capsule dimensions are not as relevant as for systems using a completely liquid core requiring micrometer sized capsules to prevent diffusion limitations into the spheroid core. Based on this, basic laboratory equipment is sufficient to produce capsules in contrast to expensive techniques, such as electro spraying, coaxial nozzles, and microfluidics [24].

Although the variances in shapes and sizes are larger than for these more controlled, automated processes, quite consistent results were achieved regarding the sizes and shapes of the capsules, considering a manual extrusion process. Simple adaptions of our current protocol, such as integration of a syringe pump for automated dispense, would not only improve the size distribution, but it would also facilitate the scaling-up of the technique.

All experiments, after deciding to continue with CMC and XG, focused on differences between the two core materials on capsule characteristics and most importantly, cell viability and growth. Whereas the capsule shape and material stiffness of the shell material may not have much impact on the cellular behavior inside, these metrics are crucial when transitioning the capsules into dynamic cultivation systems. Previous reports have indicated that with 3D suspension cultures of MSCs, reactor geometry, impeller shape, stirring speed, and cell seeding density all need to be carefully balanced to find the sweet spot where cells can assemble, the system is sufficiently agitated but cells do not experience damage by shear stress [7,48,49]. In other capsule systems using murine embryonic stem cells, it was already shown that the shell provided protection from shear stress compared to non-encapsulated spheroids [12,23]. Permeability of the capsule shell was tested with 4 kDa FITC-labelled dextran as the majority of nutrients and metabolites for cell survival are below this limit. As previously reported, alginate allowed for diffusion of the polysaccharide through the shell, a MW cutoff of 40–70 kDa was reported [34,36]. Measurements were taken at ambient temperature and in static conditions, a further increase is to be expected at physiological temperature and dynamic conditions.

Most importantly, the capsule system did allow for cell survival and proliferation. An attenuated proliferation had been previously reported for MSCs maintained in spheroids [37,38], but next to maintaining a high viability, we also confirmed actively proliferating cells after the initial condensation phase. A 2.5-fold increase in cell numbers was achieved over the four days of cultivation. Due to the nature of the surgery for donor-tissue retrieval (re-laparotomy after caesarian section), all cells used in this study are of female origin. Reumann et al. [50] and Yang et al. [51] investigated the influence of donor age, gender, and BMI and found no significant differences in the proliferative behavior of adMSCs from male and female donors. Nevertheless, the influence of donor gender and tissue origin has to be considered in future applications of the technique. Metabolic activity measured by resazurin-based assay did not correspond to the increase in cell numbers. The decrease of metabolic activity in spheroid cultures of MSCs has previously been seen by Bijonowski et al. [52]; however, the underlying mechanism is not completely resolved. The incentive for applying a resazurin-based assay had been to integrate an easy, non-invasive method to monitor cell growth. From these results, however, dissolving of capsules and cell aggregates for counting, proves to be a more reliable determination of the actual proliferation rate than the resazurin kit measurements. The retrieval of the cells out of the capsules is additionally a prerequisite for using them in a variety of cell based-therapies, either as single cells or directly as 3D aggregates. Similarly, the use of hPL throughout all the experiments was done in an effort to present a humanized, serum-free system already at this stage. Whether the presented cultivation set-up is also compatible with the still often used fetal bovine serum (FBS) supplementation needs yet to be evaluated. However, in line with good manufacturing practice (GMP) and clinical compliance, the use of xenogeneic supplements, such as FBS in cell-based products, is to be avoided. In addition, as discussed by Burnouf et al. [53], it has been demonstrated in a multitude of trials that hPL outperforms FBS in MSC and various other cell cultivation set-ups. Next to scientific reasoning, the replacement of FBS is also desired, considering ethical and animal-welfare aspects [54].

Looking at the two different thickening agents CMC and XG, used to tune the core properties, no apparent advantage regarding cell viability or proliferation could be seen. However, considering at their geometry, the more spherical shape, higher stiffness, and improved core diffusion of CMC capsules, they present more desirable traits for further transition into dynamic cultivation systems. Moreover, the slightly lower cost of the cellulose derivative, as well as its non-bacterial origin, should be considered as advantages of this system.

To summarize, we were able to develop a simple and easy to reproduce a core–shell alginate capsule production protocol for the encapsulation of adMSCs, with consideration of future scale-ups in dynamic cultivation systems. Focus was placed on cost-effective, widely available reagents and equipment, and a simple and robust production protocol. Secondly, mechanical and diffusive properties were investigated. Proliferation of adMSCs within the capsules was verified and the cell recovery out of the capsules was established, paving the way for large-scale production of physiological-like MSCs for cell-based therapies.

## Data Availability

All data from this study are available from the authors upon request.
